# Budesonide and Surfactant Therapy Versus Surfactant Alone on Incidence of Lung Disease in Preterm Infants (BEST Lung): Study Protocol for a Systematic Review and Individual Participant Data Meta‐Analysis With Nested Prospective Meta‐Analysis

**DOI:** 10.1111/apa.70638

**Published:** 2026-06-14

**Authors:** James X. Sotiropoulos, Brett J. Manley, Sol Libesman, Namasivayam Ambalavanan, Abhik Das, Christopher J. D. Mckinlay, Nalinikanta Panigrahy, Melinda Cruz, David Nguyen, Jonathan Williams, Peter Davis, Kylie E. Hunter, Anna Lene Seidler

**Affiliations:** ^1^ NHMRC Clinical Trials Centre, Faculty of Medicine and Health University of Sydney Sydney New South Wales Australia; ^2^ Department of Obstetrics, Gynaecology and Newborn Health The University of Melbourne Melbourne Victoria Australia; ^3^ Mercy Perinatal The Mercy Hospital for Women Melbourne Victoria Australia; ^4^ Clinical Sciences Murdoch Children's Research Institute Melbourne Victoria Australia; ^5^ Division of Neonatology, Department of Pediatrics University of Alabama at Birmingham Birmingham Alabama USA; ^6^ Social, Statistical and Environmental Sciences Unit RTI International Rockville Maryland USA; ^7^ Department of Paediatrics: Child and Youth Health University of Auckland Auckland New Zealand; ^8^ Rainbow Children's Hospital Hyderabad Telangana India; ^9^ NICU Lived Network Sydney New South Wales Australia; ^10^ Newborn Research The Royal Women's Hospital Melbourne Victoria Australia; ^11^ Department of Child and Adolescent Psychiatry University Medical Centre Rostock Rostock Germany; ^12^ German Centre for Child and Adolescent Health (DZKJ), Partner Site Greifswald/Rostock Rostock Germany

**Keywords:** bronchopulmonary dysplasia, budesonide, individual participant data meta‐analysis, steroids, surfactant

## Abstract

**Introduction:**

Bronchopulmonary dysplasia (BPD) is a leading cause of morbidity and mortality in preterm infants. Combining surfactant with budesonide has been proposed to prevent BPD, but results from existing randomised controlled trials (RCTs) are conflicting. Individual participant data meta‐analysis (IPD‐MA) synthesises raw trial data, increasing precision, improving assessment of data quality and enabling subgroup analyses. This IPD‐MA aims to examine the effect of surfactant plus budesonide versus surfactant alone.

**Methods:**

We will systematically search medical databases and trial registers for RCTs enrolling preterm infants born at < 32 weeks' gestation randomised to intratracheal surfactant alone or surfactant plus budesonide. Studies with major quality concerns will be excluded. Risk of bias will be assessed using Cochrane's RoB 2 tool. Two‐stage fixed‐effects IPD‐MA will be performed with co‐primary outcomes of all‐cause mortality at hospital discharge and BPD at 36 weeks' postmenstrual age. Subgroups will be examined using treatment‐covariate interactions. Certainty of evidence will be assessed using GRADE. A nested prospective meta‐analysis will also be conducted. The study is preregistered on PROSPERO (CRD42023456941).

**Conclusion:**

If effective, surfactant plus corticosteroid would be a low‐cost addition to standard care to reduce BPD and increase survival. IPD‐MA will allow further insight into treatment effects.

## Summary

1. Combining the intratracheal administration of surfactant to preterm infants with an anti‐inflammatory corticosteroid (i.e., budesonide) has been proposed to reduce bronchopulmonary dysplasia (BPD), but existing trials have conflicting results.
2. Individual participant data meta‐analysis (IPD‐MA) will allow exploration of treatment effects through increased precision, alongside enhanced assessment of data quality and subgroups
3. If effective, surfactant plus corticosteroid would be a low‐cost addition to standard care to improve outcomes.

**Abbreviations**
AD: aggregate data
BiB: budesonide in babies
BPD: bronchopulmonary dysplasia
CI: confidence interval
CNKI: Chinese National Knowledge Infrastructure
FiO_2_
: fraction of inspired oxygen
GEE: generalised estimating equations
GRADE: grading of recommendations, assessment, development and evaluations
HREC: Human Research Ethics Committee
ICMJE: International Committee of Medical Journal Editors
IPD: individual participant data
IPD‐MA: individual participant data meta‐analysis
NNT: number needed to treat
NNTH: number needed to harm
PATH: predictive approaches to treatment effect heterogeneity
PPROM: prolonged prelabour preterm rupture of membranes
PRISMA: preferred reporting items for systematic reviews and meta‐analyses
RCT: randomised controlled trial
RD: risk difference
ROB: risk of bias
RR: risk ratio
WHO ICTRP: World Health Organisation International Clinical Trials Registry Platform

## 1 Introduction

Bronchopulmonary dysplasia (BPD) is a leading cause of long‐term morbidity in children born preterm, particularly in those born extremely preterm (< 28 weeks' gestation), where its incidence is higher than 50% [1, 2]. BPD is the chronic lung disease of prematurity that evolves due to a myriad of inflammatory changes in the underdeveloped lung [3]. A diagnosis of BPD confers an increased risk of mortality [4], recurrent hospital admissions [5], childhood respiratory infections [6], asthma‐like syndromes [7], poor growth [8], neurodevelopmental impairment [9] and significantly higher healthcare related costs [10]—posing a major burden on families and health systems. Despite improvements in survival for infants born even at the extremes of viability, the incidence of BPD has increased [11], underscoring the urgent need for effective preventive strategies.

Surfactant therapy is a mainstay in the respiratory management of preterm infants. Surfactant is a complex mixture of phospholipids and proteins which functions to keep small air sacs open, increasing the surface area for gas exchange to occur [12]. Surfactant is synthesised by specialised lung cells (type II pneumocytes) which only reliably mature around 32 weeks' gestation [13]. The administration of exogenous surfactant to infants with respiratory distress syndrome is a routine and proven therapy that reduces the risk of death and BPD [14]. Like surfactant, corticosteroid medications are commonly given to preterm infants at varying points in their hospital stay. In addition, when given antenatally to pregnant people at risk of preterm birth, they reduce infant mortality and respiratory morbidity [15, 16]. In the early post‐natal period, systemic corticosteroids reduce the risk of BPD; however, this may occur at the cost of an increased risk of intestinal perforation and cerebral palsy [17]. Combining surfactant treatment with corticosteroids (e.g., budesonide) delivered directly to the lung parenchyma soon after birth has been proposed as a new method to decrease the destructive inflammatory response in the preterm lung and prevent the development of BPD, without exposure to the risks and off‐target effects of systemic administration [18].

To date, results of several randomised controlled trials (RCT) have been conflicting on whether this approach is effective. In a recent traditional aggregate data meta‐analysis of 26 RCTs with a total of 2701 preterm infants comparing surfactant plus budesonide (inhaled or intratracheal) versus surfactant alone, combined therapy reduced the risk of BPD (RR 0.61, 95% CI 0.51–0.73). A possible increased effect for infants with more severe disease was observed, but formal subgroup analyses were not possible [19]. Due to concerns with risk of bias, heterogeneity and data quality, the certainty of evidence was very low. These results were not replicated in the largest study to date, the PLUSS trial, that was not included in the aggregate data meta‐analysis. This RCT enrolled infants born at < 28 weeks' gestation randomised regardless of FiO_2_ requirement, and found no differences in the primary outcome of survival free from BPD or other short‐term secondary outcomes between the surfactant plus intratracheal budesonide group and the surfactant alone (adjusted risk difference 2.7%, 95% CI −2.1%–7.4%) [20]. Preliminary signals in exploratory analyses of this study suggest that different results and heterogeneity across existing RCTs may be due to variation in treatment effects by subgroups (e.g., infants with more severe lung disease). Similarly, in the budesonide in babies (BiB) trial of 641 preterm infants born at < 29 weeks' gestation, surfactant plus budesonide did not reduce the primary outcome of BPD or death, after stopping early for prespecified futility criteria [21].

Individual participant data meta‐analyses (IPD‐MA) are the gold‐standard approach to allow harmonisation of studies and exploration of whether the treatment works differently across subgroups. IPD‐MA is an methodology by which the raw line‐by‐line data from each of the included studies in a systematic review are collated and synthesised [22, 23]. This allows more precise statistical analyses by adjusting for covariates at the participant level, a greater examination of data quality and integrity and a more granular examination of differential treatment effects according to key subgroups. Applied to this research question, IPD‐MA may yield additional information to elucidate the effect of surfactant and steroid therapy on the outcomes of preterm infants. This includes whether differential effects exist according to participant level characteristics, such as gestational length and the severity of lung disease (e.g., FiO_2_ requirement at randomisation), and allow increased confidence in evidence through more thorough assessment of risk of bias and data quality.

### 1.1 Objectives and Hypotheses

We aim to (1) evaluate the effects of surfactant plus corticosteroid medication delivered to very preterm infants (born < 32 weeks' gestation) and (2) assess whether differential effects exist across subgroups.

We hypothesise that surfactant plus corticosteroid will reduce the incidence of BPD and death, and improve other neonatal outcomes, compared to surfactant alone. We hypothesise that surfactant plus corticosteroid will provide additional benefit to infants with more severe lung disease (e.g., higher FiO_2_ requirement at randomisation, without antenatal steroid coverage, requiring invasive ventilation at time of randomisation or with chorioamnionitis or prolonged preterm rupture of membranes), and who are more immature at birth (e.g., with shorter gestation, male sex [24] or born small for gestational age).

## 2 Methods

We will conduct a systematic review and individual participant data meta‐analysis of RCTs in preterm infants born at < 32 weeks' gestation, comparing surfactant combined with corticosteroid to surfactant alone.

The study protocol is reported in line with PRISMA‐P [25] (Appendix S1), and PRISMA‐IPD, where applicable [26]. The results will be reported in line with PRISMA 2020 [27] and the PRISMA‐IPD extension [26]. The study is registered on the international prospective register of systematic reviews (PROSPERO, CRD42023456941).

### 2.1 Ethical Considerations

Ethical approval has been granted from the University of Sydney Human Research Ethics Committee (HREC) (2025/HE000344) for a waiver of consent model for secondary use of deidentified RCT data. The primary aims of the study will align with the eligible original RCTs, and the original RCTs will have sought consent or an appropriate waiver of consent during their enrolment process. Where required by the original trial or their institution, additional approval for data re‐use will be sought from local ethics committees. Only de‐identified information will be used, ensuring participant privacy is maintained [28]. Results of the secondary analyses will be communicated to the original trials for dissemination to the participants.

### 2.2 Sources of Information

We will conduct a systematic search of medical databases (MEDLINE, Embase, Embase Classic, CENTRAL, Chinese National Knowledge Infrastructure [CNKI]) and clinical trial registers [29] (WHO ICTRP, clinicaltrials.gov) from inception to the date of searching. As the expected additional yield is low CINAHL will not be searched [30]. Searches will be updated periodically (i.e., every 12 months). Two reviewers will screen all records independently, with conflicts resolved through consensus or the introduction of a third reviewer when needed. The full search strategy is available in Appendix S2.

### 2.3 Inclusion Criteria

Eligible studies will be RCTs or cluster RCTs. Quasi‐randomised (i.e., studies using non‐random methods of participant allocation such as date of birth or day of the week) and observational studies will be excluded. By nature of the intervention being a discrete episode of drug administration rather than a prolonged period of administration, cross‐over studies are not feasible and will be excluded. Both published and unpublished studies will be included. There will be no language restrictions. Small studies (i.e., with < 50 total participants) increase heterogeneity [31] and the risk of bias [32] while providing a minimal contribution of additional information [33] (see power calculations in Appendix S3), and will be excluded. Due to changes in clinical practice related to surfactant administration over time, studies published or completed (if unpublished) before year 2000 will be excluded. Studies not meeting sufficient threshold for quality after rigorous assessment will be excluded (see Section 2.7.4).

Eligible participants will be preterm infants born at < 32 weeks' completed gestation who have received no more than one dose of surfactant and have not received any dose of postnatal corticosteroids at the time of randomisation. No restriction will be placed on infants born as part of a multiple pregnancy, or whether infants have or have not received a course of antenatal corticosteroids. Infants will be included regardless of the FiO_2_ or mode of respiratory support at the time of randomisation. Eligible participants will be treated in a neonatal unit where very preterm infants are cared for, without restriction to geographical location.

The intervention group will receive intratracheal surfactant plus corticosteroid medication administered via thin‐catheter or endotracheal tube. The comparator group will receive surfactant therapy alone (i.e., without corticosteroid) or combined with a placebo. The first dose in both groups will be administered before 48 h of age. There will be no restriction to the type (e.g., bovine or porcine) or brand (e.g., CUROSURF, Survanta or Exosurf) of surfactant used. There will be no restriction to the type of corticosteroid used, however, we expect most eligible studies will use budesonide. Interventions of surfactant plus corticosteroid delivery by methods other than thin‐catheter or endotracheal tube, such as aerosolized surfactant, oropharyngeal surfactant or inhalation of corticosteroid, will be excluded. There will be no restrictions on the dose of surfactant or corticosteroid used. We will explore possible dose–response relationships, and differential effects caused by different steroid preparations in sensitivity analyses.

### 2.4 Outcomes

Outcomes were selected in reference to the neonatal core outcome set [34] and in consultation with a patient/public representative (see Section 2.16). The co‐primary outcomes will be the diagnosis of BPD at 36 weeks' postmenstrual age or at hospital discharge (whichever is earlier), defined as the need for supplemental oxygen or positive pressure ventilation, and all‐cause hospital mortality at hospital discharge. Key secondary outcomes will include the duration of any respiratory support and discharge on home oxygen. All outcomes, including other secondary outcomes, are defined in Table 1.

**TABLE 1:** Preferred outcome definitions and timepoints.

Outcome	Preferred definition	Assessment timepoint
**Primary outcomes**		
Bronchopulmonary dysplasia	Need for supplemental oxygen or respiratory support	At 35 + 0 to 36 + 6 weeks' postmenstrual age (ideally 36 + 0)
Death	All‐cause mortality	Prior to discharge home from hospital or latest available mortality data before hospital discharge
**Key secondary outcomes**		
Duration of respiratory support	Total duration (total hours) of all positive pressure respiratory support (invasive and non‐invasive) but not including oxygen therapy	Prior to discharge home from hospital. *Infants that die before discharge will be excluded from this outcome
Discharge home with oxygen	Discharge with home oxygen therapy	At discharge home from hospital
**Other secondary outcomes**		
Survival with or without bronchopulmonary dysplasia	Ordinal outcome of: Death (all‐cause mortality) Survival with bronchopulmonary dysplasia Survival free of bronchopulmonary dysplasia	At 35 + 0 to 36 + 6 weeks' postmenstrual age (ideally 36 + 0); If unavailable, at hospital discharge
Severe intraventricular haemorrhage	Grade III or IV intraventricular haemorrhage diagnosed with imaging	Prior discharge home from hospital
Any intraventricular haemorrhage	Any intraventricular haemorrhage diagnosed with imaging	Prior discharge home from hospital
Severe brain injury on imaging	Grade III or IV intraventricular haemorrhage and/or cystic periventricular haemorrhage and/or cerebellar haemorrhage other than punctate and/or severe white matter injury on MRI	Prior discharge home from hospital
Retinopathy of prematurity treated with intraocular injections or surgery	Any retinopathy of prematurity treated with intraocular injections or surgery	Prior discharge home from hospital
Necrotising enterocolitis	Clinically or radiographically diagnosed necrotising enterocolitis	Prior discharge home from hospital
Spontaneous intestinal perforation	Spontaneous intestinal perforation diagnosed clinically or radiographically	Prior discharge home from hospital
Necrotising enterocolitis/spontaneous intestinal perforation/intestinal obstruction treated with surgery	Any necrotising enterocolitis/spontaneous intestinal perforation/intestinal obstruction treated with surgery	Prior discharge home from hospital
Patent ductus arteriosus treated with medical or surgical therapy	Patent ductus arteriosus treated with medical and/or surgical therapy	Prior discharge home from hospital
Duration of intubation	For those infants intubated, the duration of intubation and mechanical ventilation (total hours)	Prior to initial hospital discharge. *Infants that die before discharge will be excluded from this outcome
Duration of supplemental oxygen	Total duration of supplemental oxygen (total hours)	Prior discharge home from hospital. *Infants that die before discharge will be excluded from this outcome
Air leak/pneumothorax procedurally treated	Clinically or radiographically diagnosed air leak procedurally treated	Prior discharge home from hospital
Systemic postnatal corticosteroid administration	Systemic postnatal corticosteroid administration for BPD mitigation	Prior discharge home from hospital
Severe bronchopulmonary dysplasia	Grade 2 BPD (nasal cannula greater than 2 L/min or noninvasive positive airway pressure) or Grade 3 BPD (invasive mechanical ventilation) [35]	At 35 + 0 to 36 + 6 weeks postmenstrual age (ideally 36 + 0)
Severity of bronchopulmonary dysplasia	Ordinal outcome according to Jensen 2019 criteria [35]: Grade 0 (No BPD) Grade 1 (Mild BPD with low flow nasal canula 2 L/min or less) Grade 2 (Moderate BPD with low flow nasal canula > 2 L/min or non‐invasive respiratory support) Grade 3 (Severe BPD with invasive mechanical ventilation)	At 35 + 0 to 36 + 6 weeks postmenstrual age (ideally 36 + 0)
Pulmonary haemorrhage	Occurrence of pulmonary haemorrhage as determined by treating clinician/defined in individual study	Prior discharge home from hospital
Hospital length of stay	Total length of stay in hospital (days)	At discharge home from hospital
**Follow up outcomes at early childhood**		
Death or major neurodevelopmental/neurosensory disability	Death or major neurodevelopmental/neurosensory disability as defined below	At 12 to 36 months of age. Preferred assessment at 24 months
Major Neurodevelopmental/neurosensory disability	Cerebral palsy with Gross Motor Function Classification System (GMFCS) level 2 or higher Developmental delay more than 1.3 standard deviations (< 10th centile) below population means. (Preferred scale Bayley Scales of Infant Development [BSID] Fourth edition). If BSID‐4 is not available we will use data from another standardised developmental test (e.g., BSID‐III or BSID‐II) Severe visual impairment with acuity 6/60 or worse, or as defined in trial Deafness requiring amplification, or as defined in trial	
All‐cause mortality at follow‐up	Death from any cause	
Cerebral palsy	Any cerebral palsy	
Developmental delay	Developmental delay more than 1.3 standard deviations (< 10th centile) below population means in cognitive, language, social–emotional or adaptive domains. (Preferred scale Bayley Scales of Infant Development [BSID] Fourth edition). If BSID‐4 is not available we will use data from another standardised developmental test (e.g., BSID‐III or BSID‐II) or a screening tool (e.g., ASQ‐3)	
Motor delay	Motor delay more than 1.3 standard deviations (< 10th centile) below population means. (Preferred scale BISD‐4 motor domains). IF BSID‐4 is not available we will use data from another standardised developmental test (e.g., BSID‐III or BSID‐II) or a screening tool (e.g., ASQ‐3)	
Emotional‐behavioural difficulty	Clinical diagnosis of autism spectrum disorder (ASD), attention deficit hyperactivity disorder (ADHD) or *z*‐score more than 1.3 standard deviations (< 10th centile) below population means on standardised emotional‐behavioural instrument	
Bayley Scales of Infant Development (continuous scales)	Individual components of BSID‐4 or BSID‐3 (cognition, language, motor, social–emotional, adaptive behavioural)	
Cognitive impairment (continuous scale)	Any standardised developmental test. Data from each test will be standardised by dividing by the standard deviation of the trial	
Language impairment (continuous scale)		
Motor impairment (continuous scale)		
Number of hospitalisations for respiratory illnesses	Number of discrete hospital admissions for respiratory illnesses	At 12–36 months age. Preferred assessment at 24 months
Incidence of wheezing episodes	Number of wheezing episodes (both with and without hospital admission)	
Body size	Sex‐ and corrected age‐standardised scores for height, weight, head circumference and body mass index	
Parent reported child quality of life	Parent report child quality of life. Instruments will be standardised across trials	

### 2.5 Outcome Harmonisation

Where appropriate, variable and outcome definitions, methods of assessment and time points of assessment will be harmonised across trials to enable synthesis of similar or equivalent data. Variable/outcome harmonisation refers to using IPD to reconstruct available data in accordance with meta‐analysis specific definitions with respect to time point of assessment, measurement tool, format/unit and method of aggregation. Where outcome harmonisation is not appropriate (e.g., if too much scientific content or granularity would be lost), we will revert to study specific definitions of outcomes and comment on the implications of this in the review.

### 2.6 Governance and Collaboration Management

The governance plan (see Appendix S4) outlines the structure of the BEST Lung Collaboration. The steering committee will be comprised of clinical, methods and statistical experts (J.X.S. [co‐chair], A.L.S. [co‐chair], K.E.H., B.J.M., S.L.). The steering committee will be responsible for oversight of review processes, conducting the systematic search, data processing and checking, and statistical analyses. Each eligible trial will be invited to nominate at least one (but no more than two) study representatives to join the collaboration advisory group. Advisory group members will have input on key design aspects of the protocol and statistical analysis plan, interpretation of results, and will critically appraise and provide feedback on study documents. Collaboration members meeting ICMJE authorship requirements will be invited to be a co‐author on relevant study publications.

### 2.7 Data Management

#### 2.7.1 Data Collation and Extraction

Data will be sourced from original trial investigators, or online repositories. We will make no fewer than three attempts (spaced 14 days apart) to contact the corresponding author of each of the eligible studies with a request to begin the process of data sharing. The primary method of communication will be via email. If after three attempts, we are unable to establish contact we will attempt to contact authors by other means (i.e., using social media platforms, via professional networks or by contacting authors other than the corresponding author). If despite exhaustive attempts, we are unable to establish communication with a trial representative, we will mark the study as ‘individual participant data not available—uncontactable’.

For all studies (including those that provide IPD), we will extract study level meta‐data including information about inclusion/exclusion criteria of the individual study, study design, year, country, setting, total number of participants and information about the enrolling centre(s). For studies that provide IPD, we will request IPD for all outcomes (see Table 1) as well as key demographic information (e.g., gestational age at birth, birthweight, sex, antenatal steroid status), case report forms (if required to resolve uncertainties within the IPD) and associated documentation including protocols, data codebooks or manuals of procedure. For studies that do not provide IPD, we will extract outcome and demographic data from published study reports. A full list of data items to be requested/extracted is available in Appendix S5.

For the extraction of study level information, or outcome information for studies not providing IPD, data will be extracted by two independent reviewers in duplicate using a standardised data extraction form designed for this study. Discrepancies will be resolved by consensus or by adding a third review author. Any uncertainties will be resolved by attempting to contact study investigators or by consulting relevant supporting study documents such as protocols or trial registration records.

#### 2.7.2 Data Storage

Data transfer will occur via secure‐file transfer with password protected files in accordance with the University of Sydney *Research Data Management Procedures 2015*. The data will be hosted on a secure database at the University of Sydney and will be stored in accordance with *Research Data Management Policy 2014*. Only authorised users will have access to the data.

#### 2.7.3 Data Processing and Checking

On receipt of IPD, the data will be checked and recoded into common format for use in meta‐analyses. Data will be checked with respect to range, validity, internal consistency, consistency with published records and missing items by one data analyst and checked independently by a second team member. Any discrepancies will be resolved via queries with the relevant trial representative.

#### 2.7.4 Data Quality Checks

We will use *The IPD Integrity* tool to assess the data quality of the received IPD [36]. Checks will be conducted for retractions, provision of IPD, communication, ethics, registration, randomisation, plausibility, data patterns, baseline characteristics, correlations, date violations, patterns of allocation and internal and external inconsistencies [36]. Data quality assessments will be performed by two independent reviewers, with conflicts resolved via a third, or by consensus with the steering committee if required. Studies with no concerns in any domain will be included. Studies with minor concerns that can be resolved via communication/clarification with trial representatives will be included. For studies with minor concerns that cannot be resolved through communication with trial investigators, decisions regarding inclusion in the main and/or sensitivity analyses will be made on a case‐by‐case basis in consultation with the steering committee and in accordance with a decision tree [36]. These decisions will be guided by structured methodological and clinical judgement, considering the totality of concerns across all domains and their circumstantial nature. Any study with major quality concerns will be excluded. Any decisions to exclude a study because of quality concerns will be made by consensus with the steering committee.

#### 2.7.5 Risk of Bias and Data Integrity in the Individual Studies

Risk of bias (ROB) will be assessed using the Cochrane ROB‐2 tool [37]. We will use IPD to inform ROB assessments following published guidance [38]. For example, we will use IPD to test and examine the randomisation sequence and examine attrition bias in more detail. Using IPD to inform ROB assessments has been shown to reduce uncertainty in ROB assessments [38]. Assessments will be conducted in duplicate by two independent reviewers, with disagreements resolved by consensus or the introduction of a third reviewer.

### 2.8 Statistical Analysis

Statistical analyses will be conducted using the open‐source software, R. We will calculate risk ratio (RR) for dichotomous outcomes and mean or rate differences for continuous outcomes along with 95% confidence intervals (CI). Risk difference (RD) with 95% CI will be calculated based on estimates of baseline risk of outcomes in the study population. Number needed to treat (NNT) or number needed to harm (NNTH) will be reported for statistically significant outcomes. Where possible, analyses will be by intention‐to‐treat. The primary estimands are defined as follows: for the treatment of intratracheal surfactant plus corticosteroid compared to the control intratracheal surfactant alone, in the population of preterm infants < 32 weeks' gestation that survive to 36 weeks' postmenstrual age (or to discharge if discharged prior to 36 weeks), including any infants that may have experienced treatment discontinuation due to any reason (i.e., intention to treat), the change in risk of BPD at 36 weeks' postmenstrual age summarised as a risk ratio, and in the population of preterm infants < 32 week's gestation, including any infants that may have experienced treatment discontinuation due to any reason (i.e., intention to treat), the change in risk of all‐cause mortality at discharge summarised as a risk ratio.

We will perform two‐stage individual participant data meta‐analyses. In the first stage, we will estimate the treatment effects and standard errors within each trial. To do this, we will use generalised estimating equations (GEE) with an exchangeable working correlation structure to account for the clustering of multiples from the same pregnancy/cluster RCTs. Models will be fitted with a log link from the binomial family for dichotomous outcomes and identity link for continuous outcomes. This analysis will use the geeglm function in the geepack package [39]. The link and model for continuous outcomes will be selected dependent on the distribution of the data (e.g., identity link if normally distributed, gamma if severe positive skew and positive). Continuous outcomes will also be examined for zero inflation. Effects and standard errors are then combined in a standard meta‐analysis model in the second stage [40]. Pooling absolute effect from binary outcomes may be biased by varying baseline risk across settings [41], instead, we will estimate absolute effects of these outcomes by transforming the pooled relative effect into absolute estimates using the assumed baseline risk for control. This will be done using simulation to account for baseline uncertainty [42]. Due to the small number of expected studies, we anticipate the estimation of the heterogeneity parameter to be unreliable and will thus use common (fixed) effect models for our main analyses [43]. We will explore random effects in sensitivity analyses. However, if the number of studies is large, we will consider using random effects for the primary analysis and pre‐specify this in the statistical analysis plan before analyses are undertaken.

If models converge and provided we have adequate power per parameter within each trial, we will adjust for effect modifiers/prognostic variables including gestational age at birth (completed weeks), infant sex, small for gestation age (< 10th centile) and antenatal corticosteroid coverage (no antenatal corticosteroids/any antenatal corticosteroids). If models do not converge, we will iteratively remove variables. If we are unable to adjust for all covariates, we will prioritise adjusting for gestational age.

In random effect models we will calculate between study variance and report prediction intervals (if there are an adequate number of trials to reliably estimate heterogeneity parameters) as measures of heterogeneity [44]. *I* ^2^ will be reported as a measure of the total proportion of variability due to between study heterogeneity [44].

Missing data will be handled in accordance with the principles outlined by Jakobsen et al. [45]. If the proportion of missing data is negligible (i.e., < 5%) we will conduct complete case analyses, if it is large (i.e., > 40%) we will consider removing that trial and comparing in sensitivity analyses. Otherwise, if the underlying assumptions are appropriate, multiple imputation will be used. Only covariates will be imputed for the primary analyses. We will evaluate the effect of imputing the outcomes in a sensitivity analysis if there is > 5% missing primary outcome data. The implications of missing data will be discussed in the review.

### 2.9 Sensitivity Analyses

Prespecified sensitivity analyses for the co‐primary outcomes include random effects analysis; assessing the effects of missing data and/or imputation of missing outcome data; exploring one‐stage models in the event of small studies or a small number of events; exploring the implications of combining aggregate data (AD) with IPD; exploring alternate outcome definitions/outcome harmonisation decisions if appropriate; leave‐one‐out analyses; excluding studies with high risk of bias in one or more domains; excluding studies with minor/borderline quality concerns. Sensitivity analyses will only be conducted for the co‐primary outcomes, unless there are specific concerns for other outcomes requiring investigation.

### 2.10 Subgroup Analyses

We will explore possible differential effects of treatment across potential effect modifiers. At the individual‐level these will be FiO_2_ at time of randomisation, FiO_2_ ≥ 0.5 at time of randomisation, mode of respiratory support at time of randomisation (invasive/non‐invasive), any surfactant given prior to randomisation, gestational age, small for gestational age status (< 10th Fenton centile for birthweight), antenatal corticosteroid coverage (no antenatal corticosteroid/any antenatal corticosteroid), chorioamnionitis (defined separately as clinical, histological, and either clinical or histological), prolonged prelabour preterm rupture of membranes (PPROM, > 24 h) and infant sex.

To explore whether treatment is effective across higher and lower resourced settings, we will perform subgroup analysis according to the perinatal mortality rate of the country in which the infant received care. Treatment effect could possibly vary across ethnic groups (which may be due to factors including socioeconomic status, cultural differences, genetics and/or other factors); we will perform subgroup analysis according to ethnicity at the individual participant level to explore this. If there are insufficient data on ethnicity, we will use non‐majority ethnicity (as defined by the trial setting) as an alternative definition. These subgroup analyses will only be performed if sufficiently granular data are available to ensure appropriate and sensitive reporting of results [46]. To explore the effect of socioeconomic deprivation on treatment effects, we will perform subgroup analyses according to the components of socioeconomic status (education, occupation, income). If insufficient data are available, we will use maternal education as a surrogate for socioeconomic status. Subgroup analyses will also be performed according to the immigration status of parents as an additional psychosocial determinant of health.

If we identify studies that use corticosteroid other than budesonide, we will also conduct subgroup analyses by type of corticosteroid at the trial level. If we identify multiple doses of surfactant plus budesonide in the eligible studies, or if some trials allow for repeated administrations of budesonide, we will explore dose response relationships. Subgroup analyses will only be conducted for the primary outcomes of BPD and all‐cause mortality at hospital discharge. We will conduct subgroup analyses for other outcomes if a specific hypothesis is prespecified in the Statistical Analysis Plan. Hypotheses for all pre‐specified subgroup analyses will be reported in the Statistical Analysis Plan.

To avoid inappropriate borrowing of across trial information in subgroup analyses (potentially causing ecological bias), we will estimate treatment‐covariate interactions in each trial separately in stage one, and then synthesise them in stage two of the individual participant data meta‐analysis. To visualise the data for binary subgroups, we will summarise the subgroup effects using the floating subgroup package that avoids aggregation bias [47]. For continuous variables, if model fit statistics such as residuals indicate linear models do not fit and analyses are feasible with the number of eligible trials, non‐linear models will be explored using restricted cubic splines [48].

If data are appropriate, we will consider developing a clinical prediction model to explore heterogenous treatment effects using a risk or effect modelling approach in line with the PATH statement in future studies [49]. These methods will be described separately.

### 2.11 Nested Prospective Meta‐Analysis

The two largest known studies [20, 21, 50] agreed to collaborate and conduct an IPD‐MA prospectively (i.e., prior to their results being known while both studies were recruiting) and registered this planned prospective meta‐analysis on PROSPERO (CRD42023456941). This approach satisfies the definition of a prospective meta‐analysis [51], which are advantageous to improve data harmonisation and reduce publication bias [52]. In addition to primary analyses with other eligible studies, these two studies (and any other ongoing studies with results not known at time of searching, but with IPD available at the time of analysis) will be analysed separately as part of a nested prospective meta‐analysis. The nested prospective meta‐analysis will only include the primary and key secondary outcomes. Additional studies that are not completed at time of analysis may be considered for future cycles of analyses.

### 2.12 Combining Individual Participant Data With Aggregate Data

We expect some eligible trials may not provide individual participant data. It has previously been demonstrated that studies that do not provide IPD may be at higher risk of bias, have lower methodological quality and have more integrity concerns [53, 54, 55]. Using AD also limits the ability to perform more complex analyses, including an examination of effect modification across key subgroups.

We will follow prespecified rules to decide whether to combine aggregate data (AD) with IPD for the primary analysis including examination of proportion of AD, statistical methods in AD studies, effect sizes, risk of bias and data quality. These rules are adapted from previous IPD meta‐analyses conducted in paediatrics and neonatology (Appendix S6) [53, 54, 56]. This process will be further pre‐specified in the statistical analysis plan, and reported as a supplement to the results publication.

### 2.13 Publication Bias

If the number of studies is sufficient (i.e., ≥ 10) we will explore publication and other potential bias with a funnel plot and Egger's test [57]. However, the potential effects of publication bias will be minimised by including a search of unpublished evidence (i.e., searching clinical trial registers) [29], and the nested prospective meta‐analysis component for the two largest studies (see Section 2.11).

### 2.14 Confidence in Cumulative Evidence

Certainty of evidence will be appraised using the GRADE framework [58]. Certainty of evidence appraisals will be conducted in duplicate by two independent reviewers. Conflicts will be resolved via consensus or with the introduction of a third reviewer if required.

### 2.15 Handling Potential Conflicts of Interest

In line with Cochrane Handbook guidance for managing potential conflicts of interest [59], original study authors will not be involved in processes related to data processing, extraction, checking, risk of bias assessment, certainty of evidence assessment or other review processes for their own study.

### 2.16 Patient and Public Involvement

We will work with a public/patient representative (MC) who has lived experience of neonatal intensive care to ensure the study aims, outcomes and interpretation reflect what matters most to families. This includes input into protocol development (including outcome selection), interpretation of results and the use of parent‐friendly language. We plan to co‐develop plain language resources to share with families once the study is complete.

## 3 Discussion

Interventions to decrease rates of BPD are urgently required. Adding intratracheal corticosteroid to the routine administration of surfactant would be a low‐cost addition to standard care if found to be effective in reducing BPD and other preterm related morbidity. The advantages of intratracheal administration of steroid in preterm infants may include minimising the toxicities associated with systemic postnatal corticosteroids (i.e., increased risk of cerebral palsy) [35]. Budesonide, for example, has minimal systemic absorption (approximately 4%) but potent local anti‐inflammatory effects [60, 61]. Budesonide may also form intracellular deposits in epithelium, prolonging local action [62]. Previous studies have established that mixing surfactant formulations with budesonide does not disturb its tensile properties [60].

Individual participant data meta‐analysis will allow thorough examination of data quality, integrity, risk of bias and exploration of potential subgroup effects [40]. However, we anticipate a few possible limitations. If studies choose not to share IPD we may be underpowered for some outcomes. We will aim to mitigate this through comprehensive data sharing requests, and the planned analysis with IPD and aggregate data combined. Even if all studies share IPD, we expect data availability for some domains may be low; as such, some subgroup analyses may not be feasible. Any analyses that are not performed will be outlined in the statistical analysis plan or results publication.

The results of this systematic review and individual participant data meta‐analysis and nested prospective meta‐analysis will be published in a peer reviewed journal and presented at relevant conferences upon completion. We will aim to collaborate with guideline bodies to ensure rapid uptake of findings into clinical practice. Results are expected in 2027.

## Author Contributions

**Anna Lene Seidler:** conceptualization, methodology, project administration, resources, supervision, writing – review and editing. **Brett J. Manley:** conceptualization, methodology, funding acquisition, writing – review and editing, supervision. **Christopher J. D. Mckinlay:** methodology, writing – review and editing. **Namasivayam Ambalavanan:** methodology, writing – review and editing. **David Nguyen:** methodology, writing – review and editing, resources, project administration. **Abhik Das:** methodology, writing – review and editing. **Melinda Cruz:** methodology, writing – review and editing. **Sol Libesman:** methodology, writing – review and editing, resources, supervision. **Nalinikanta Panigrahy:** methodology, writing – review and editing. **Kylie E. Hunter:** methodology, writing – review and editing, conceptualization, supervision, resources, project administration. **James X. Sotiropoulos:** conceptualization, methodology, funding acquisition, writing – original draft, writing – review and editing, project administration, resources. **Peter Davis:** methodology, writing – review and editing. **Jonathan Williams:** methodology, writing – review and editing, resources, project administration.

## Funding

J.X.S. is supported by an NHMRC Postgraduate Research Scholarship (GNT2031240) for aspects of this work. J.X.S. holds a Thrasher Research Fund Early Career Award (2025) for this project. B.J.M. is supported by an NHMRC Investigator Grant L1 (GNT2016662) for aspects of this work. K.E.H. is supported by an NHMRC Investigator Grant EL1 (GNT2040943) for aspects of this work. N.A. is supported by grants from the National Institutes of Health (R01HL156275, R01HL157256, UG1HD034216). The funders had no role in the design of the study.

## Ethics Statement

Ethical approval has been granted from the University of Sydney Human Research Ethics Committee (HREC) (2025/HE000344) for a waiver of consent model for secondary use of deidentified RCT data.

## Conflicts of Interest

The authors declare no conflicts of interest. Trial representatives (B.J.M., C.J.D.M., N.A., A.D., N.P.) and advisors (P.D.) were involved in randomised controlled trials expected to be eligible for this IPD‐MA. These authors will not be involved in screening eligible studies, data quality and integrity assessments or risk of bias assessments for their own studies.

## Supporting information


**Appendix S1:** PRISMA‐P Checklist.

**Appendix S2:** Search strategy.

**Appendix S3:** Power calculations.

**Appendix S4:** BEST Lung Study governance plan.

**Appendix S5:** BEST Lung Study data items and codebook.

**Appendix S6:** Rules to guide decision whether to combine aggregate data with individual participant data.

## Data Availability

Requests for re‐use of the combined BEST Lung database from external sources will be considered through a moderated access process. All requests will be reviewed by the steering group, who will communicate with each trial representative. If the request for use of the combined dataset is approved, the combined BEST Lung database will be shared. Only requests for data use related to scientifically sound and ethically approved projects will be considered. Requests for commercial purposes will not be approved. As trial representatives remain the custodians of the individual participant data from their trial, individual trials can opt out of sharing data.
