# Supplementary material for: Budesonide and Surfactant Therapy Versus Surfactant Alone on Incidence of Lung Disease in Preterm Infants (BEST Lung): Study Protocol for a Systematic Review and Individual Participant Data Meta‐Analysis With Nested Prospective Meta‐Analysis
Source: Acta Paediatr. 2026 Jun 14;115(10):2078–90. doi: 10.1111/apa.70638 (PMC13552583; doi:10.1111/apa.70638)
Supplement: Supplementary file 1 [file APA-115-2078-s001.docx]

**Appendix 2. Search strategy**

**Medline (via OVID)**

PARTICIPANTS

1. Exp infant, newborn or Intensive Care, Neonatal/ or Intensive Care Units, Neonatal/ or Gestational Age/
2. (babe or babes or baby* or babies or gestational age? Or infant? Or infantile or infancy or low birth weight or neonat* or neo-nat* or newborn* or new born? Or newly born or premature or pre-mature or pre-matures or prematures or prematurity or pre-maturity or preterm or preterms or pre term? Or preemie or preemies or premies or premie or VLBW or VLBWI or VLBW-I or VLBWs or LBW or LBWI or LBWs or ELBW or ELBWI or ELBWs or NICU or NICUs).ti,ab,kw,kf.
3. Bronchopulmomary dysplasia/ or BPD.mp
4. 1 or 2 or 3

INTERVENTION

5. Pulmonary Surfactants/
6. LISA.mp.
7. MIST.mp.
8. (Less invasive surfactant administration).mp
9. (Minimally invasive surfactant therapy).mp
10. Exogenous surfactant
11. (Curosurf or Exosurf or Survanta or Infasurf or Surfaxin).mp
12. (Poractant alfa or Beractant or Calfactant or Lucinactant).mp
13. Budesonide/ or Glucocorticoids
14. (budesonide or steroid or corticosteroid*).mp
15. Corticosteroid
16. Adrenal Cortex Hormones
17. 5 or 6 or 7 or 8 or 9 or 10 or 11 or 12
18. 13 or 14 or 15 or 16
19. 17 AND 18

RCT FILTER

20. exp randomized controlled trial/
21. controlled clinical trial.pt.
22. randomized.ab.
23. placebo.ab.
24. drug therapy.fs.
25. randomly.ab.
26. trial.ab.
27. groups.ab.
28. [20-27]OR
29. exp animals/ not humans.sh.
30. 28 not 29

FINAL

31. 4 AND 19 AND 3

**Embase and Embase Classic (via Ovid)**

PARTICIPANTS

1. hospitalized infant/ or high risk infant/ or infant disease/ or infant/ or infant mortality/ or newborn intensive care/ or gestational age/ or low birth weight/ or very low birth weight/
2. (babe or babes or baby* or babies or gestational age? or infant? or infantile or infancy or low birth weight or neonat* or neo-nat* or newborn* or new born? or newly born or premature or pre-mature or pre-matures or prematures or prematurity or pre-maturity or preterm or preterms or pre term? or preemie or preemies or premies or premie or VLBW or VLBWI or VLBW-I or VLBWs or LBW or LBWI or LBWs or ELBW or ELBWI or ELBWs or NICU or NICUs).ti,ab,kw,kf.
3. lung dysplasia/ or neonatal respiratory distress syndrome/
4. (bronchopulmonary dysplasia) ti,ab,kw,kf.
5. 1 or 2 or 3 or 4

INTERVENTION

6. lung surfactant/ or surfactant/ or lung surfactant extract/ or artificial lung surfactant/ or poractant/
7. (LISA or MIST or less invasive surfactant administration or minimally invasive surfactant administration or exogenous surfactant).mp.
8. (Curosurf or Exosurf or Survanta or Infasurf or Surfaxin).mp
9. (Poractant alfa or Beractant or Calfactant or Lucinactant).mp
10. budesonide/ or corticosteroid/ or glucocorticoid/
11. (budesonide or steroid or corticosteroid*).mp
12. 6 or 7 or 8 or 9
13. 10 or 11
14. 12 AND 13

RCT FILTER

15. exp randomized controlled trial/
16. controlled clinical trial/random$.ti,ab.
17. randomization/
18. intermethod comparison/
19. placebo.ti,ab.
20. (compare OR compared OR comparison).ti,ab.
21. ((evaluated OR evaluate OR evaluating OR assessed OR assess) AND (compare OR compared OR comparing OR comparison)).ab
22. (open adj label).ti,ab.
23. ((double OR single OR doubly OR singly) adj (blind OR blinded OR blindly)).ti,ab.
24. double blind procedure/
25. parallel group$1.ti,ab.
26. (crossover OR cross over).ti,ab.
27. ((assign$ OR match OR matched OR allocation) adj5 (alternate OR group$1 OR intervention$1 OR patient$1 OR subject$1 OR participant$1)).ti,ab.
28. (assigned OR allocated).ti,ab.
29. (controlled adj7 (study OR design OR trial)).ti,ab.
30. (volunteer OR volunteers).ti,ab.
31. human experiment/
32. trial.ti.
33. or/15-32
34. (random$ adj sampl$ adj7 ("cross section$" OR questionnaire$1 OR survey$ OR database$1)).ti,ab. NOT (comparative study/ OR controlled study/ OR randomi?ed controlled.ti,ab. OR randomly assigned.ti,ab.)
35. cross-sectional study/ NOT (exp randomized controlled trial/ OR controlled clinical trial/ OR controlled study/ OR randomi?ed controlled.ti,ab. OR control group$1.ti,ab.)
36. (((case adj control$) and random$) not randomi?ed controlled).ti,ab.
37. (systematic review.ti,ab. NOT (trial OR study).ti)
38. (non random$ NOT random$).ti,ab.
39. "random field$".ti,ab.
40. (random cluster adj3 sample$).ti,ab.
41. (review.ab. AND review.pt.) NOT trial.ti.
42. ("we searched".ab. AND (review.ti. OR review.pt.))
43. "update review".ab.
44. (databases adj4 searched).ab.
45. (rat OR rats OR mouse OR mice OR swine OR porcine OR murine OR sheep OR lambs OR pigs OR piglets OR rabbit OR rabbits OR cat OR cats OR dog OR dogs OR cattle OR bovine OR monkey OR monkeys OR trout OR marmoset$1).ti. AND animal experiment/
46. animal experiment/ NOT (human experiment/ OR human/)
47. or/34-46
48. 33 NOT 47

FINAL

49. 5 AND 14 AND 48

**EBM Reviews – Cochrane Central Register of Controlled Trials (via OVID)**

PARTICIPANTS

1. Infant, Small for Gestational Age/ or Infant, Extremely Low Birth Weight/ or Infant/ or Infant, Newborn/ or Infant, Newborn, Diseases/ or Infant, Premature, Diseases/ or Infant, Very Low Birth Weight/ or Infant, Extremely Premature/ or Infant Care/ or Incubators, Infant/ or Nurseries, Infant/ or Infant Mortality/ or Infant, Premature/ or Infant Death/
2. (babe or babes or baby* or babies or gestational age? or infant? or infantile or infancy or low birth weight or neonat* or neo-nat* or newborn* or new born? or newly born or premature or pre-mature or pre-matures or prematures or prematurity or pre-maturity or preterm or preterms or pre term? or preemie or preemies or premies or premie or VLBW or VLBWI or VLBW-I or VLBWs or LBW or LBWI or LBWs or ELBW or ELBWI or ELBWs or NICU or NICUs).ti,ab,kw,kf.
3. Bronchopulmonary Dysplasia/ or Respiratory Distress Syndrome, Newborn/
4. 1 or 2 or 3

INTERVENTION

5. Pulmonary Surfactants/
6. Surface-Active Agents/
7. (LISA or MIST or less invasive surfactant administration or minimally invasive surfactant treatment).mp.
8. (Curosurf or Exosurf or Survanta or Infasurf or Surfaxin).mp.
9. (Poractant alfa or Beractant or Calfactant or Lucinactant).mp.
10. Budesonide/
11. Adrenal Cortex Hormones/
12. Glucocorticoids/
13. (budesonide or steroid or corticosteroid*).mp.
14. 5 or 6 or 7 or 8 or 9
15. 10 or 11 or 12 or 13

FINAL

16. 14 AND 15
17. 4 AND 16

**WHO ICTRP (Clinical Trial Register)**

*Search 1 (Advanced)*

[CONDITION]

(preterm OR premature OR respiratory distress syndrome OR BPD OR bronchopulmonary dysplasia OR chronic lung disease)

AND [INTERVENTION]

((surfactant OR curosurf OR survanta OR Exosurf OR MIST or LISA OR INSURE) AND (budesonide OR corticosteroid OR steroid))

Recruitment status: ALL

*Search 2 (Advanced)*

[TITLE]

((surfactant OR curosurf OR survanta OR Exosurf OR MIST or LISA OR INSURE) AND (budesonide OR corticosteroid OR steroid))

[CONDITION]

(preterm OR premature OR respiratory distress syndrome OR BPD OR bronchopulmonary dysplasia OR chronic lung disease)

Recruitment status: ALL

*Search 3 (Basic)*

(preterm OR premature OR respiratory distress syndrome OR BPD OR bronchopulmonary dysplasia OR chronic lung disease) AND ((surfactant OR curosurf OR survanta OR Exosurf OR MIST or LISA OR INSURE) AND (budesonide OR corticosteroid OR steroid))

**Clinicaltrials.gov (Clinical Trial Register)**

[Other terms/Expert search]

(preterm OR premature OR respiratory distress syndrome OR BPD OR bronchopulmonary dysplasia OR chronic lung disease) AND ((surfactant OR curosurf OR survanta OR Exosurf OR MIST or LISA OR INSURE) AND (budesonide OR corticosteroid OR steroid))

**Chinese National Knowledge Infrastructure (CNKI.net)**

Advanced search

Subject: 表面活性剂 AND Subject: 布地奈德(budesonide)

Synonym search: TRUE
