# Supplementary material for: Budesonide and Surfactant Therapy Versus Surfactant Alone on Incidence of Lung Disease in Preterm Infants (BEST Lung): Study Protocol for a Systematic Review and Individual Participant Data Meta‐Analysis With Nested Prospective Meta‐Analysis
Source: Acta Paediatr. 2026 Jun 14;115(10):2078–90. doi: 10.1111/apa.70638 (PMC13552583; doi:10.1111/apa.70638)
Supplement: Supplementary file 2 [file APA-115-2078-s002.docx]

**Appendix 3. Power calculations**

**Power calculations performed with “metapower” package for R**

Based on the 2 large studies that, to date, have committed to data sharing (with total 1659 participants) we have 81% power with fixed-effects models (with two-tailed significant at the 5% level and conservative heterogeneity assumptions, I^2^ of 25%) to detect effect sizes as small as 0.07.

Based on other recent systematic reviews and meta-analyses, we expect at least 9 studies to be eligible with total sample size ~2600. If data are received for all these studies, we will have 95% power for fixed effects and 85% power for random effects models to detect effect sizes as small as 0.07 (again, with two-tailed significance at the 5% level and conservative heterogeneity assumptions, I^2^ of 25%)_._

To demonstrate the minimal impact small studies (n=50 or less) have on these power calculations, if data for only the 2 large studies with 1659 participants and 1 further study with 50 participants are received, the power to detect effect sizes of 0.07 increases from 81% to only 82%.
