# Supplementary material for: Budesonide and Surfactant Therapy Versus Surfactant Alone on Incidence of Lung Disease in Preterm Infants (BEST Lung): Study Protocol for a Systematic Review and Individual Participant Data Meta‐Analysis With Nested Prospective Meta‐Analysis
Source: Acta Paediatr. 2026 Jun 14;115(10):2078–90. doi: 10.1111/apa.70638 (PMC13552583; doi:10.1111/apa.70638)
Supplement: Supplementary file 3 [file APA-115-2078-s003.docx]

**BEST Lung Study Governance Plan**

**Document History**

Initial Draft – (23^rd^ of June 2025)
Revision 1 – (4^th^ of August 2025)
Finalised – (17^th^ of September 2025)

**Prepared by:**

James Sotiropoulos, Jonathan Williams, Kylie Hunter, Anna Lene Seidler on behalf of the BEST Lung Collaboration

The governance of the BEST Lung Collaboration is shown in the Table below, accurate as at 28/02/2025.

**Table 1. Governance structure of the BEST Lung Collaboration**

| **Steering group** | | |
| --- | --- | --- |
| Co-chairs: James Sotiropoulos, Anna Lene Seidler  Methods expert: Kylie Hunter Clinician expert: Brett Manley Statistician: Sol Libesman | | |
| **Collaboration members** | | |
| Trial representatives | | |
| Nayasamivan Ambalavan, Abhik Das  Christopher JD McKinlay | | |
| Project and data management team | Patient/public representative | Advisors |
| Jonathan Williams  David Nguyen | Melinda Cruz | Peter Davis |

**Steering Group**
The BEST Lung Steering Group comprises five members who hold overall responsibility for the project. The co-chairs will lead the day-to-day research activities involved in the collaboration, develop agendas, and chair meetings. The other steering group members will provide relevant expertise and advice on a regular basis. The steering group will make final decisions on key design elements including statistical analysis and results interpretation with input from the wider collaboration. The steering group will aim to meet every 3-6 weeks, with meeting frequency adjusted as required.

**Trial representatives**
The primary role of trial representatives is to ensure their trial is appropriately represented in the BEST Lung Collaboration. Each eligible trial will be invited to nominate at least one trial representative to join the collaboration. Trial representatives will be required to liaise with the steering group and project/data management team to coordinate the data sharing process for their eligible trial – this includes sharing de-identified individual participant data, and related study materials (e.g., meta-data, codebooks, study protocols). Trial representatives will be expected to communicate with the steering group and project/data management team to answer queries during data processing and recoding. They will have the opportunity to provide input on the protocol (if joined before the protocol is finalised), statistical analysis plan and final manuscript, if they wish. There will usually be only one Representative per trial, but in certain instances two representatives are permissible. This will be decided on a case-by-case basis. Potential scenarios for two representatives to be invited include: a.) A representative is about to retire and nominates an additional representative that will lead the trial in future b.) The trial is led by a junior researcher (e.g. PhD student) but initiated and supervised by a more senior researcher c.) The trial is co-led by two investigators that make all major decisions collaboratively

**Project and data management team**
The project and data management team will be based at the University of Sydney, NHMRC Clinical Trials Centre. These team members will be supervised by steering group members to conduct key review processes including systematic searches, contacting eligible studies and coordinating data sharing, data processing/checking/recoding, risk of bias and data quality assessments, and statistical analyses.

**Advisors**
Advisors are invited to join the collaboration by the steering group due to their relevant expertise (i.e., clinical or methodological). Advisors will be expected to provide input on the protocol, statistical analysis plan and interpretation of results. In instances where a trial wishes to nominate more than one trial representative, one investigator may be nominated as the trial representative with a secondary investigator nominated as an advisor. See above criteria to consider multiple representatives.

**Patient/public representative**
The patient/public representative will be invited to review and provide input on the study design (protocol and statistical analysis plan) and results interpretation (main results manuscript) with respect to the views of someone with lived experience of the research topic. The patient/public representative will work with the steering group to ensure the voices of those with lived experience are reflected throughout aspects of the work, and in the communication of the findings.

**Communication**
Regular communication will be in the form of email. Newsletter updates will be sent periodically. There will be whole collaboration meetings at least yearly, or more frequently if required when approaching key deadlines. Face-to-face meetings and informal meetings will be arranged at relevant conferences.

**Publication and Dissemination Policy**
The collaboration will publish the protocol and the results manuscript in an appropriate peer reviewed journal. All collaboration members will be invited to review and critically appraise draft documents for publication. The standard timeframe to provide feedback on study documents will be 15 business days.

**Authorship policy**
For the protocol and main results manuscript all active collaboration members will be invited to be named authors. We refer to the NHMRC Authorship Criteria (see [Authorship: A guide supporting the Australian Code for the Responsible Conduct of Research](https://www.nhmrc.gov.au/sites/default/files/documents/attachments/Authorship-Guide.pdf)), the International Committee of Medical Journal Editors guidance on authorship (see [ICMJE | Recommendations | Defining the Role of Authors and Contributors](https://www.icmje.org/recommendations/browse/roles-and-responsibilities/defining-the-role-of-authors-and-contributors.html)) and the role definitions outlined above. We interpret “*Substantial contributions to the conception or design of the work; or the acquisition, analysis, or interpretation of data for the work”* to be met if collaboration members satisfactorily complete their duties as described in the role descriptions as above (including the facilitation of data sharing for trial representatives). All authors will also be required to review publication drafts and approve the final version. For every publication, named authorship and order will be proposed and agreed upon by the collaboration in the early stages of the project. If necessary, authorship order can be changed after discussion with the collaboration based on respective contributions. Depending on the number of authors, group authorship (i.e., *on behalf of the BEST Lung Collaboration)* may be considered.

**Other projects**
The methods developed and data curated by the collaboration may provide opportunities for side projects and publications. These may include additional analyses and methods development work. Working groups for these projects will be formed, and those making substantial contributions will be invited to be named authors on relevant side publications. Other collaboration members will be recognized with group authorship (i.e., *on behalf of the BEST Lung Collaboration*).

**Confidentiality**
All members of the BEST Lung Collaboration will be required to keep the result of the BEST Lung Study, and any related projects, strictly confidential until a time that they are made publicly available (i.e., after journal publication or presentation to the public at a relevant conference).

**Conflicts of Interest**
BEST Lung Collaboration members will be required to declare any conflicts of interest related to this body of work to the BEST Lung Steering Group. If additional conflicts of interest arise through the course of the program of work, collaboration members will be expected to update the steering group as soon as possible.

To mitigate potential conflicts of interest related to study assessment and data-analysis, collaboration members will not contribute to risk of bias/data quality assessments for their own trial (with the exception of answering queries raised by the assessors).

**Intellectual Property**
The trial representatives retain custodianship/ownership of the individual participant data for their respective trials. The BEST Lung collaboration will obtain permission to use and modify the data for the purposes of the BEST Lung study and related projects. Any scientific outputs (e.g., the results of the individual participant data meta-analyses, methods projects, and side projects) will be the intellectual property of the BEST Lung Collaboration.

**Data Sharing**
Requests for re-use of the combined BEST Lung database from external will be considered through a moderated access process. All requests will be reviewed by the steering group, who will communicate with each trial representative. If the request for use of the combine dataset is approved, the combined BEST Lung database will be shared. Only requests for data use related to scientifically sound and ethically approved projects will be considered. Requests for commercial purposes will not be approved. As trial representatives remain the custodians of the individual participant data from their trial, individual trials can opt out of sharing data.
