# Supplementary material for: Budesonide and Surfactant Therapy Versus Surfactant Alone on Incidence of Lung Disease in Preterm Infants (BEST Lung): Study Protocol for a Systematic Review and Individual Participant Data Meta‐Analysis With Nested Prospective Meta‐Analysis
Source: Acta Paediatr. 2026 Jun 14;115(10):2078–90. doi: 10.1111/apa.70638 (PMC13552583; doi:10.1111/apa.70638)
Supplement: Supplementary file 5 [file APA-115-2078-s005.docx]

**Appendix 6. Rules to guide decision whether to combine aggregate data with individual participant data**

1. We will consider the proportion of participants without IPD available to determine whether adding the AD could impact the results.
2. We will examine the statistical methods (i.e., did the study control for similar covariates), study characteristics (i.e., are there systematic differences between the AD and IPD studies), and baseline participant characteristic (i.e., did AD and IPD studies assess comparable populations).
3. We will compare effect sizes for the primary outcome, where large differences in effect sizes between AD and IPD studies might suggest significant bias or differences in study populations.
4. We will compare risk of bias and trustworthiness/data integrity assessments between IPD and AD studies.

The final decision whether to combine IPD with AD will be made via consensus with the steering committee.

If concerns about the data availability outweigh any concerns about systematic bias/trustworthiness concerns in the AD, we will combine IPD with AD for the primary analysis. Otherwise, AD will not be included in the primary analysis, and combined AD and IPD analyses will be reported as a sensitivity analysis for the primary and key secondary outcomes. If we do combine AD with IPD, we will also conduct sensitivity analyses for all outcomes using IPD alone. Aggregate data will not be combined with IPD for any of the prespecified subgroup analyses.
