# Supplementary material for: Budesonide and Surfactant Therapy Versus Surfactant Alone on Incidence of Lung Disease in Preterm Infants (BEST Lung): Study Protocol for a Systematic Review and Individual Participant Data Meta‐Analysis With Nested Prospective Meta‐Analysis
Source: Acta Paediatr. 2026 Jun 14;115(10):2078–90. doi: 10.1111/apa.70638 (PMC13552583; doi:10.1111/apa.70638)
Supplement: Supplementary file 6 [file APA-115-2078-s006.docx]

**Appendix 5 – BEST Lung Study Data Items and Codebook**

**Study Level Information**

| **Variable Name** | **Description** | **Suggested coding** |
| --- | --- | --- |
| rand_unit | Unit of randomisation used  *If ‘other’, provide information in rand_unit_other variable below* | 1=Individual;  2=Cluster (e.g. health centre); 3=Other |
| rand_unit_other | Brief description of the unit of randomisation used | Free text |
| country1 | Country/countries of recruitment  *If >1 country, provide as separate variables, i.e. country2, country3, etc.* | Free text |
| mortality_rate | Perinatal mortality rate of recruitment country (deaths per 1000 live births). | Whole number |
| mult_eligible | Were multiples (eg twins, triplets) eligible for your study? | 0=No, only singleton eligible;  1=Yes, eligible |
| mult_rand | For multiples, were infants randomised individually or together?  *Option 1 if mother was unit of randomisation.* | 1=Multiples randomised together;  2=Multiples randomised separately |
| n_infants | Number of infants included in your study | Whole number |
| enrol_start | Date of first participant enrolment | dd/mm/yyyy |
| enrol_end | Date of last participant enrolment | dd/mm/yyyy |
| consent_cat | Type of consent/assent for participation | 1=Waiver of consent (IRB/ethics committee waived the requirement to obtain any informed consent from participants);  2=Deferred consent (randomisation occurred without participant's knowledge, then informed consent was requested at a later phase);  3=Informed consent or assent (obtained prospectively prior to randomisation) |
| consent | Describe consent/assent procedures | Free text |
| group1 | Provide your trial’s classification or brief description of intervention group, e.g. intratracheal surfactant plus corticosteroid medication.  *If > 1 intervention group, describe under variables group2, group3, etc.* | Free text |
| control | Provide your trial’s classification or brief description of control group, i.e. intratracheal surfactant only. | Free text |
| steroid_type1 | Type of corticosteroid used in intervention group1 (e.g. budesonide).  *If > 1 intervention group, describe under variables* steroid_type2*,* steroid_type3*, etc.* | Free text |
| steroid_dose1 | Dosage of corticosteroid used in intervention group1 (include the unit of measurement, e.g. mg/kg).  *If > 1 intervention group, describe under variables* steroid_dose2*,* steroid_dose3*, etc.* | Free text |
| surfactant_brand | Brand name of surfactant used in trial (e.g. CUROSURF®, Survanta®, Exosurf™). | Free text |
| surfactant_type | Type of surfactant used in trial (e.g. bovine or porcine). | Free text |
| surfactant_dose | Dosage of surfactant used in trial (include the unit of measurement, e.g. mg/kg) | Free text |
| steroid_repeat_allowed | Were repeated doses of corticosteroid allowed in your study? | 0=No; 1=Yes |
| inclusion_criteria | Describe all inclusion criteria of infants in your study, e.g. preterm infants born at <32 week’s completed gestation | Free text |
| exclusion_criteria | Describe all exclusion criteria of infants in your study, e.g. have not received any dose of postnatal corticosteroids at time of randomisation | Free text |

**Pregnancy/Parental Baseline Information at Randomisation**

| **Variable Name** | **Description** | **Suggested coding** |
| --- | --- | --- |
| mat_dob | Maternal date of birth. | dd/mm/yyyy |
| mat_age | Maternal age. | Years |
| mat_ethnicity | Ethnicity of mother.  *If data for father is available, add pat_ethnicity.* | Ethnicity variables will be harmonised across the available studies depending on data granularity and availability. |
| mat_ethnicity_broad | Ethnicity of mother, defined into broader categories.  *If data for father is available, add pat_ethnicity_broad.* | 1=Dominant ethnicity;  2=Non-dominant ethnicity/mixed |
| mat_education | Mother’s highest education level.  *If data for father is available, add pat_education.* | 1= Primary school education  2= Completed primary school, did not complete high school;  3=High school graduate;  4=Non-university tertiary education or qualification or incomplete university;  5=University graduate or postgraduate |
| mat_employment | Mother’s employment status.  *If data for father is available, add pat_employment.* | Using coding below OR trialist classification* OR free text  1=Full time employment (including paid leave);  2=Part time employment (including paid leave);  3=Unemployed (includes retired, student without employment, unpaid leave, home duties, charity work) |
| mat_occupation | Level of mother’s most recent occupation.  *If data for father is available, add pat_employment.* | Using coding below OR trialist classification* OR free text  1=Manager; 2=Professional/associate; 3=Tradesperson/farmer; 4=Clerical, sales and services; 5=Labourer; 6=Homemaker/housewife; 7=Other (including unemployed) |
| mat_immigration | Was mother born in study country or outside study country?  *If data for father is available, add pat_immigration.* | 1=Born in study country;  2=Born outside study country |
| mat_income | Amount/level of mother’s income.  *If data for father is available, add pat_income.* | Using trialist classification* OR free text |
| household_income | Total household income per year (as defined in your trial*).  *Average income will be adapted to each country and time of recruitment due to changes in average income over time.* | 1=Low income (below average income);  2=Moderate income (average income);  3=High income (above average income) |
| parity | Number of times the mother has previously given birth at gestational age >20 weeks (or as defined in your trial*). | Whole number |
| gravidity | Number of times the mother has previously been pregnant. | Whole number |
| previous_preg | Has the mother had any previous pregnancy?  *Only required if above variables parity and gravidity were not collected.* | 0=No; 1=Yes |
| EDD | Estimated date of delivery | dd/mm/yyyy |
| antenatal steroid | Level of antenatal steroid coverage | 0 = None  1 = Any antenatal steroid coverage (including incomplete) |
| antenatal_steroid_detailed | Level of antenatal steroid coverage with more sublevels (as defined in your trial*). | 0=None;  1=Incomplete;  2= Complete |
| labour | Spontaneous onset of labour. | 0=No; 1=Yes |
| delivery_mode | Mode of delivery. | 1=Normal vaginal delivery;  2=Instrumental vaginal delivery;  3=Caesarean before onset of  labour;  4=Caesarean after onset of labour |
| delivery_mode_broad | Mode of delivery, defined into broader categories. | 1=Vaginal delivery;  2=Caesarean delivery |
| multiple | Is the current pregnancy a multiple pregnancy? | 0=No (singleton);  1=Yes (multiple) |
| PPROM | Prolonged premature rupture of membranes (>24 hours, or as defined in your trial*). | 0=No; 1=Yes |
| Chorio_any | Chorioamnionitis (clinical, histological or both | 0=No; 1=Yes |
| Chorio_clinical | Chorioamnionitis (clinical diagnosis) | 0=No; 1=Yes |
| Chorio_histological | Chorioamnionitis (histological diagnosis) | 0=No; 1=Yes |
| chorio_method | If only one variable for chorioamnionitis was provided please provide the variable “chorio_other” and code here what method of chorioamnionitis diagnosis was used.  *If ‘other’, provide information in chorio_method_other variable below.* | 1 = Either clinical or histological  2=Clinical diagnosis  3=Histological diagnosis  4=Other diagnosis method  5=Unclear diagnosis method |
| chorio_method_other | Brief description of the diagnosis method used to detect chorioamnionitis | Free text |

**Infant Baseline Information at Randomisation**

| **Variable Name** | **Description** | **Suggested coding** | |
| --- | --- | --- | --- |
| infant_ID | Unique infant identifier used within your trial. | | Anonymised, no patient names, can be character or number |
| twin_ID | Unique identifier of twin/triplet etc if born as part of multiple pregnancy. | | See infant_ID |
| centre | Unique identifier for study centre in which randomisation occurred. | | Trialist classification* (if available) or free text |
| rand_date | Date of randomisation. | | dd/mm/yyyy |
| inf_dob | Infant date of birth. | | dd/mm/yyyy |
| inf_inborn | Infant born at study centre in which randomisation occurred (inborn).  *Infants born in one setting, usually non-tertiary NICU, and retrieved to higher resourced setting should be categorised as ‘No’ (outborn).* | | 0=No (outborn);  1=Yes (inborn) |
| ethnicity | Ethnicity of infant.  *Can be the same as mat_ethnicity if not available.* | | Ethnicity variables will be harmonised across the available studies depending on data granularity and availability. |
| ethnicity_broad | Ethnicity of infant, defined into broader categories. | | 1=Dominant ethnicity  2=Non-dominant ethnicity/mixed |
| group | Indicate to which group the participant was allocated | | 1 = control group (intratracheal surfactant WITHOUT corticosteroid)  2 = intervention group (intratracheal surfactant WITH corticosteroid) *Add additional groups if present in your study* |
| compliance | Compliance with allocated intervention (as defined in your trial*). | | Free text |
| GA_weeks | Gestation at birth, using best estimate (weeks)  *Enter completed weeks here and completed days below. Eg: 36+4 would be GA_weeks=36; GA_days=4* | | Completed weeks |
| GA_days | Gestation at birth, using best estimate (days)  *Enter completed days here and completed weeks above. Eg: 36+4 would be GA_weeks=36; GA_days=4* | | Completed days |
| sex | Infant sex | | 1=Male  2=Female  3=Intersex/ambiguous |
| birthweight | Birthweight of infant (grams) | | Whole number (grams) |
| SGA | Small for gestational age (<10^th^ Fenton centile, or as defined in your trial*) | | 0=No; 1=Yes |
| apgar_1min | Standard Apgar score at 1 minutes of life | | Whole number 0 to 10 |
| apgar_5min | Standard Apgar score at 5 minutes of life | | Whole number 0 to 10 |
| FiOx | Fraction of inspired oxygen (FiO_2_) at time of randomisation | | % |
| FiOx_greater | Fraction of inspired oxygen (FiO_2_) greater than or equal to 0.5 (50%) at time of randomisation | | 0=No; 1=Yes |
| prior_surfactant | Any surfactant given prior to randomisation | | 0=No; 1=Yes |
| resp_support | Did the infant receive any respiratory support subsequent to delivery and/or initial resuscitation? (as defined in your trial*)  *If yes, please indicate which respiratory support the infant received below.* | | 0=No; 1=Yes |
| mech_vent | Mechanical ventilation | | 0=No; 1=Yes |
| CPAP_support | Continuous positive airway pressure | | 0=No; 1=Yes |
| nasal_oxygen | Low flow nasal oxygen | | 0=No; 1=Yes |
| supp_oxygen | Supplementary oxygen, any | | 0=No; 1=Yes |
| supp_oxygen_36wk | Supplementary oxygen at 36 weeks postmenstrual age | | 0=No; 1=Yes |
| resp_other | List any other forms of respiratory support provided between birth and discharge (as defined in your trial*). | | Free text |

**Infant Follow-up Information at 36 weeks’ Postmenstrual Age (or Hospital Discharge)**

| **Variable Name** | **Description** | **Suggested coding** |
| --- | --- | --- |
| BPD | Presence of bronchopulmonary dysplasia (need for supplemental oxygen or respiratory support).  *Assessed at 35+0 to 36+6 weeks’ postmenstrual age (ideally 36+0).* | 0=No; 1=Yes |
| severe_BPD | Grade 2 BPD (nasal cannula greater than 2 L/min or non-invasive positive airway pressure) or Grade 3 BPD (invasive mechanical ventilation)  *Assessed at 35+0 to 36+6 weeks’ postmenstrual age (ideally 36+0).* | 0=No; 1=Yes |
| BPD_severity | Severity of bronchopulmonary dysplasia.  *Assessed at 35+0 to 36+6 weeks’ postmenstrual age (ideally 36+0).* | 0=No support;  1=Nasal cannula ≤2 L/min (Grade 1 BPD);  2=Nasal cannula >2 L/min or non-invasive positive airway pressure (Grade 2 BPD);  3=Invasive mechanical ventilation (Grade 3 BPD) |
| death_discharge | Infant death from any cause. *Assessed prior to discharge home from hospital.* | 0=No; 1=Yes |
| death_discharge_cause | Primary cause of infant death (as defined in your trial*).  *Assessed prior to discharge home from hospital.* | Free text |
| date_death_discharge | Date of infant death.  *Assessed prior to discharge home from hospital.* | dd/mm/yyyy |
| age_death_discharge | Age of infant death (in days).  *Assessed prior to discharge home from hospital.* | Days |
| home_oxygen | Discharge with home oxygen therapy.  *Assessed at discharge home from hospital.* | 0=No; 1=Yes |
| duration_resp_supp | Total duration of positive pressure respiratory support (invasive and non-invasive) *(*as defined in your trial*) not including low flow oxygen.  *Assessed prior to discharge home from hospital.* | Hours |
| duration_intubation | Total duration of intubation and mechanical ventilation.  *Assessed prior to discharge home from hospital.* | Hours |
| duration_oxygen | Total duration of supplemental oxygen.  *Assessed prior to discharge home from hospital.* | Hours |
| IVH | Intraventricular haemorrhage, any grade (as defined in your trial*).  *Assessed prior to discharge home from hospital.* | 0=No; 1=Yes |
| worst_IVH | Worst grade of IVH (as defined in your trial*).  *Assessed prior to discharge home from hospital.* | Free text |
| severe_IVH | Severe intraventricular haemorrhage (grade III or IV) diagnosed with imaging (or as defined in your trial*).  *Assessed prior to discharge home from hospital.* | 0=No; 1=Yes |
| PVL | Periventricular leukomalacia (as defined in your trial*).  *Assessed prior to discharge home from hospital.* | 0=No; 1=Yes |
| ROP | Any retinopathy of prematurity (ROP) (as defined in your trial*).  *Assessed prior to discharge home from hospital.* | 0=No; 1=Yes |
| severe_ROP | Stage 3 or 4 ROP, or ROP requiring medical or surgical intervention (or as defined in your trial*).  *Assessed prior to discharge home from hospital.* | 0=No; 1=Yes |
| NEC | Any necrotising enterocolitis (e.g. Bell’s stage 2 or above, *or as defined in your trial*).*  *Assessed prior to discharge home from hospital.* | 0=No; 1=Yes |
| intestinal_perforation | Spontaneous intestinal perforation (perforation not associated with necrotising enterocolitis or other known pathology, or as defined in your trial*).  *Assessed prior to discharge home from hospital.* | 0=No; 1=Yes |
| intestinal_obstruction_surgical | Intestinal obstruction treated with surgery (or as defined in your trial*).  *Assessed prior to discharge home from hospital.* | 0=No; 1=Yes |
| PDA | Patent ductus arteriosus treated with medical and/or surgical therapy *(or as defined in your trial*)*.  *Assessed prior to discharge home from hospital.* | 0=No; 1=Yes |
| pneumothorax | Clinically or radiographically diagnosed air leak (pneumothorax) procedurally treated *(*as defined in your trial*).  *Assessed prior to discharge home from hospital.* | 0=No; 1=Yes |
| postnatal_sys_steroid | Systemic postnatal corticosteroid administration for bronchopulmonary dysplasia mitigation *(*as defined in your trial*).  *Assessed prior to discharge home from hospital.* | 0=No; 1=Yes |
| pulmonary_haemorrhage | Occurrence of pulmonary haemorrhage (as defined in your trial*).  *Assessed prior to discharge home from hospital.* | 0=No; 1=Yes |
| date_hosp_discharge | Date of discharge from hospital after birth.  *Assessed at discharge home from hospital.* | dd/mm/yyyy |
| hospital_days | Total number of days in hospital after birth (including hospital transfers)  *Assessed at discharge home from hospital.* | Days |
| inf_excluded | Was the infant excluded from your analyses? | 0=No; 1=Yes |
| reason_excluded | If the infant was excluded from your analyses, please state reason (as defined in your trial*). | Free text |

**Infant Follow-up Information at Early Childhood (24±12 months of age)**

| **Variable Name** | **Description** | **Suggested coding** |
| --- | --- | --- |
| followup_any1 | Was a follow-up test conducted for this infant? (the extension “1” maps onto all instances of the 1st follow up test)  *If >1 follow-up tests were undertaken, label as followup_any2, followup_any3, etc. Note: tests may be conducted at the same timepoint as test 1, or at a later date.* | 0=No; 1=Yes |
| followup_age1 | Chronological age - What was the infant’s uncorrected age at the 1st follow-up test?  *If >1 follow-up tests were undertaken, label as followup_age2, followup_age3, etc.* | Months |
| corrected_age1 | What was the infant’s corrected age at the 1st follow-up test? Corrected age, or adjusted age, is the premature baby's chronological age minus the number of weeks or months they were born early, e.g. a one-year  *If >1 follow-up tests were undertaken, label as corrected_age2, corrected_age3, etc.* | Months |
| followup_height1 | Height of infant at 1st follow-up test  *If >1 follow-up tests were undertaken, label as followup_height2, followup_height3, etc.* | Centimetres |
| followup_weight1 | Weight of infant at 1st follow-up test  *If >1 follow-up tests were undertaken, label as followup_weight2, followup_weight3, etc.* | Kilograms |
| followup_circum1 | Head circumference of infant at 1st follow-up test  *If >1 follow-up tests were undertaken, label as followup_circum2, followup_circum3, etc.* | Centimetres |
| followup_zBMI1 | Sex- and corrected age-standardised score for body mass index z-score of infant at 1st follow-up test.  *If >1 follow-up tests were undertaken, label as followup_zBMI2, followup_zBMI3, etc.* | Numeric |
| inf_death | Infant death from any cause  *May be the same as death_discharge (i.e. 1=Yes) if infant died prior to discharge home from hospital.* | 0=No; 1=Yes |
| inf_death_cause | Primary cause of death (as defined in your trial*).  *May be the same as death_discharge_cause if infant died prior to discharge home from hospital.* | Free text |
| date_inf_death | Date of infant death.  *May be the same as date_death_discharge if infant died prior to discharge home from hospital.* | dd/mm/yyyy |
| age_inf_death | Age of infant death (in months). | Months |
| neuro_dis_any | Major neurodevelopmental /neurosensory disability, defined as any of the following:   (1) Cerebral palsy with Gross Motor Function Classification System (GMFCS) level 2 or higher. (2) Developmental delay more than 1.3 standard deviations (<10^th^ centile) below population means. (Preferred scale Bayley Scales of Infant Development [BSID] Fourth edition). If BSID-4 is not available we will use data from another standardised developmental test (e.g., BSID-III or BSID-II). (3) Severe visual impairment with acuity 6/60 or worse, or as defined in trial. (4) Deafness requiring amplification, or as defined in trial. | 0=No; 1=Yes |
| CP_any | Any cerebral palsy (as defined in your trial*). | 0=No; 1=Yes |
| CP | Cerebral palsy with Gross Motor Function Classification System (GMFCS) level 2 or higher. | 0=No; 1=Yes |
| blind | Severe visual impairment with acuity 6/60 or worse (or as defined in trial*). | 0=No; 1=Yes |
| deaf | Deafness requiring amplification (or as defined in trial*). | 0=No; 1=Yes |
| cog_comp1 | Score on cognitive composite scale at 1st follow-up test.  *If there are multiple cognition scales label cog_comp1 for test 1, cog_comp2 for test 2, etc. - the extension “1” maps onto all instances of the 1st follow-up test, “2” for the 2nd test, etc.* | Numeric;  -999 = incomplete or invalid |
| cog_comp_delay1 | Cognitive delay (as defined in your trial*).  *If there are multiple cognition scales label cog_comp_delay1 for test 1, cog_comp_delay2 for test 2, etc. - the extension “1” maps onto all instances of the 1st follow-up test, “2” for the 2nd test, etc.* | 0=No; 1=Yes |
| cog_comp_tool 1 | Name of tool used to assess cognition and type of score, e.g., BSID-4 cognition composite.  *If there are multiple cognition scales label cog_comp_tool1 for test 1, cog_comp_tool2 for test 2, etc. - the extension “1” maps onto all instances of the 1st follow-up test, “2” for the 2nd test, etc.* | Free text |
| lang_comp1 | Score on language composite scale at 1st follow-up test.  *If there are multiple language scales label lang_comp1 for test 1, lang_comp2 for test 2, etc. - the extension “1” maps onto all instances of the 1st follow up test, “2” for the 2nd test, etc.* | Numeric;  -999 = incomplete or invalid |
| lang_comp_delay1 | Language delay (as defined in your trial*).  *If there are multiple language scales label lang_comp_delay1 for test 1, lang_comp_delay2 for test 2, etc. - the extension “1” maps onto all instances of the 1st follow up test, “2” for the 2nd test, etc.* | 0=No; 1=Yes |
| lang_comp_tool 1 | Name of tool used to assess language and type of score, e.g., BSID-4 language composite.  *If there are multiple language scales label lang_comp_tool1 for test 1, lang_comp_tool2 for test 2, etc. - the extension “1” maps onto all instances of the 1st follow up test. “2” for the 2nd test, etc.* | Free text |
| motor_comp1 | Score on motor composite scale at 1st follow-up test.  *If there are multiple motor composite scales label motor_comp1 for test 1, motor_comp2 for test 2, etc. - the extension “1” maps onto all instances of the 1st follow up test, “2” for the 2nd test, etc.* | Numeric;  -999 = incomplete or invalid |
| motor_comp_delay1 | Motor delay (as defined in your trial*).  *If there are multiple motor composite scales label motor_comp_delay1 for test 1, motor_comp_delay2 for test 2, etc. - the extension “1” maps onto all instances of the 1st follow up test, “2” for the 2nd test, etc.* | 0=No; 1=Yes |
| motor_comp_tool1 | Name of tool used to assess motor and type of score, e.g., BSID-4 motor composite.  *If there are multiple motor scales label motor_comp_tool1 for test 1, motor_comp_tool2 for test 2, etc. - the extension “1” maps onto all instances of the 1st follow up test. “2” for the 2nd test, etc.)* | Free text |
| social_emotional1 | Score on social-emotional scale at 1st follow-up test.  *If there are multiple social-emotional scales label social_emotional1 for test 1, social_emotional2 for test 2, etc. - the extension “1” maps onto all instances of the 1st follow up test. “2” for the 2nd test, etc.)* | Numeric;  -999 = incomplete or invalid |
| social_emotional_delay1 | Social-emotional delay (as defined in your trial*).  *If there are multiple social-emotional scales label social_emotional_delay1 for test 1, social_emotiona_delay2 for test 2, etc. - the extension “1” maps onto all instances of the 1st follow up test. “2” for the 2nd test, etc.)* | 0=No; 1=Yes |
| social_emotional_tool1 | Name of tool used to assess social-emotional behaviour and type of score (e.g., BSID-4 social emotional composite score equivalent).  *If there are multiple social-emotional scales label social_emotiona_tool1 for test 1, social_emotional_tool2 for test 2, etc. - the extension “1” maps onto all instances of the 1st follow up test. “2” for the 2nd test, etc.)* | Free text |
| adaptive_behav1 | Score on adaptive behaviour scale at 1st follow-up test.  *If there are multiple social-emotional scales label adaptive_behav1 for test 1, adaptive_behav2 for test 2, etc. - the extension “1” maps onto all instances of the 1st follow up test. “2” for the 2nd test, etc.)* | Numeric;  -999 = incomplete or invalid |
| adaptive_behav_delay1 | Adaptive behaviour delay (as defined in your trial*).  *If there are multiple social-emotional scales label adaptive_behav_delay1 for test 1, adaptive_behav_delay2 for test 2, etc. - the extension “1” maps onto all instances of the 1st follow up test. “2” for the 2nd test, etc.)* | 0=No; 1=Yes |
| adaptive_behav_tool1 | Name of tool used to assess adaptive behaviour and type of score (e.g., BSID-4 adaptive composite score equivalent).  *If there are multiple social-emotional scales label adaptive_behav_tool1 for test 1, adaptive_behav_tool2 for test 2, etc. - the extension “1” maps onto all instances of the 1st follow up test. “2” for the 2nd test, etc.)* | Free text |
| n_hosp admissions | Number of discrete hospital admissions for respiratory illnesses (as defined in your trial*). | Whole number |
| n_wheezing | Number of wheezing episodes (both with and without hospital admission, or as defined in your trial*). | Whole number |
| qol_score1 | Parent-reported child quality of life at 1st follow-up test.  *If there are multiple quality of life scales label qol_score1 for test 1, qol_score2 for test 2, etc. - the extension “1” maps onto all instances of the 1st follow up test. “2” for the 2nd test, etc.)* | Numeric;  -999 = incomplete or invalid |
| qol_score_tool1 | Name of tool used to assess parent-reported child quality of life at 1st follow-up test.  *If there are multiple quality of life scales label qol_score_tool1 for test 1, qol_score_tool2 for test 2, etc. - the extension “1” maps onto all instances of the 1st follow up test. “2” for the 2nd test, etc.)* | Free text |
